# The prognostic value of platelet-to-lymphocyte ratio on the long-term renal survival in patients with IgA nephropathy

**DOI:** 10.1007/s11255-020-02651-3

**Published:** 2020-10-28

**Authors:** Dan Chang, Yichun Cheng, Ran Luo, Chunxiu Zhang, Meiying Zuo, Yulin Xu, Wei Dai, Yueqiang Li, Min Han, Xiaofeng He, Shuwang Ge, Gang Xu

**Affiliations:** 1 Department of Nephrology, University of Electronic Science and Technology, Sichuan Academy of Sciences and Sichuan Provincial People’s Hospital, Chengdu, Sichuan China; 2 Department of Nephrology, Tongji Hospital of Tongji Medical College of Huazhong University of Science and Technology, Wuhan, Hubei China

**Keywords:** Immunoglobulin A nephropathy, Platelet-to-lymphocyte ratio, Renal survival, Cohort

## Abstract

**Purpose:**

Platelet-to-lymphocyte ratio (PLR) was established showing the poor prognosis in several diseases, such as malignancies and cardiovascular diseases. But limited study has been conducted about the prognostic value of PLR on the long-term renal survival of patients with Immunoglobulin A nephropathy (IgAN).

**Methods:**

We performed an observational cohort study enrolling patients with biopsy-proven IgAN recorded from November 2011 to March 2016. The definition of composite endpoint was eGFR decrease by 50%, eGFR < 15 mL/min/1.73 m^2^, initiation of dialysis, or renal transplantation. Patients were categorized by the magnitude of PLR tertiles into three groups. The Kaplan–Meier curves and multivariate Cox models were performed to determine the association of PLR with the renal survival of IgAN patients.

**Results:**

330 patients with a median age of 34.0 years were followed for a median of 47.4 months, and 27 patients (8.2%) had reached the composite endpoints. There were no differences among the three groups (PLR < 106, 106 ≤ PLR ≤ 137, and PLR > 137) in demographic characteristics, mean arterial pressure (MAP), proteinuria, and estimated glomerular filtration rate (eGFR) at baseline. The Kaplan–Meier curves showed that the PLR > 137 group was significantly more likely to poor renal outcomes than the other two groups. Using univariate and multivariate cox regression analyses, we found that PLR > 137 was an independent prognostic factor for poor renal survival in patients with IgAN. Subgroup analysis revealed that the PLR remained the prognostic value for female patients or patients with eGFR less than 60 mL/min/1.73 m^2^.

**Conclusions:**

Our results underscored that baseline PLR was an independent prognostic factor for poor renal survival in patients with IgAN, especially for female patients or those patients with baseline eGFR less than 60 mL/min/1.73 m^2^.

## Introduction

Immunoglobulin A nephropathy (IgAN) is one of the most common primary glomerulonephritis worldwide [1]. The number of patients with IgAN accounts for 58.2% of glomerulonephritis in China [2]. Studies have shown that 15% to 40% of patients with IgAN develop the end-stage renal disease (ESRD) by 10 to 20 years after diagnosis [3, 4]. As one of the main reasons to increase the social burden, however, it is still challenging to precisely predict the outcomes of IgAN patients [5, 6]. Several clinical indicators such as renal function and proteinuria at biopsy are demonstrated to be associated with the renal outcome of IgAN [7–9]. But we need to find new risk factors of IgAN to timely prevent the disease from deterioration.

Platelet-to-lymphocyte ratio (PLR), calculated as platelet count divided by the lymphocyte count, is an inexpensive, replicable, and easily measurable index. There is increasing evidence suggesting that PLR may serve as a novel inflammatory marker and prognostic factors for various diseases, such as malignancies [10–13], cardiovascular diseases [14, 15], chronic obstructive pulmonary disease [16] and saphenous vein graft disease [17].

Although the pathogenesis of IgAN is not completely understood, multiple mechanisms may be involved, and inflammation is known to play a key role [18–21]. However, the clinical significance of PLR in the IgAN process remains unclear. Hence, we conducted this study to explore the prognostic value of PLR on long-term renal survival among patients with IgAN.

## Materials and methods

### Study design and study population

**Fig. 1:**
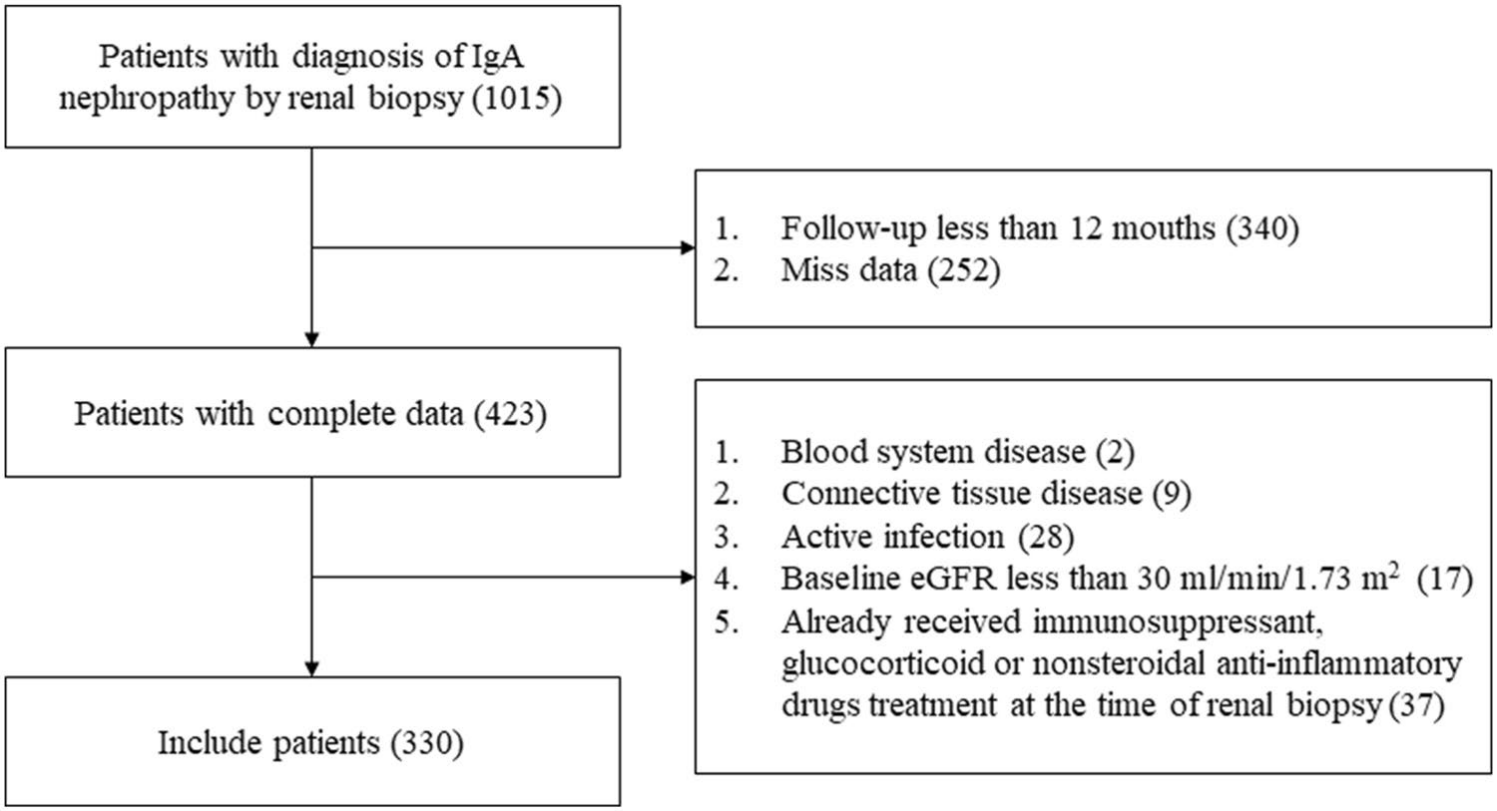
Flow chart of the patients included in the study

Three hundred and thirty patients with IgAN were recruited in the Tongji hospital affiliated to Tongji medical college of Huazhong University of Science and Technology from November 1, 2011, to March 1, 2016 (Fig. 1). We included biopsy-proven IgAN patients with complete clinical and pathological data. And we excluded patients with blood system disease, connective tissue disease, active infection, and baseline eGFR less than 30 mL/min/1.73 m^2^. Patients who received immunosuppressant, glucocorticoid, or nonsteroidal anti-inflammatory drugs treatment at the time of renal biopsy were also excluded.

### Demographic and clinical data

Baseline demographic, clinical and laboratory data were collected from all patients at the time of renal biopsy, including age, gender, systolic blood pressure (SBP), diastolic blood pressure (DBP), white blood cell count (WBC), platelet count, lymphocyte count, hemoglobin, serum albumin, serum creatinine (Scr), uric acid (UA), estimated glomerular filtration rate (eGFR), and proteinuria quantity.

PLR was calculated as the ratio of the absolute platelet count to the absolute lymphocyte count on preoperative routine blood tests.

The blood pressure of patients was measured by a cuff pressure method using a mercury sphygmomanometer or an electronic sphygmomanometer. Mean arterial pressure (MAP) was calculated by (SBP + 2 × DBP)/3.

eGFR is calculated by the Modification of Diet in Renal Disease Study (MDRD) equation: 186 × (Creatinine/88.4)^−1.154^ × (Age)^−0.203^ × (0.742 if female) × (1.210 if black) [22].

Regular visits at intervals of 6 months were performed in every patient. Urine sediment and renal function were tested at every visit throughout follow-up. All follow-up data were updated to March 1, 2019.

### Study outcomes

For survival analysis, the definition of composite endpoint was eGFR decrease by 50% or ESRD. ESRD was defined as eGFR < 15 mL/min/1.73 m^2^, initiation of dialysis, or renal transplantation. Patients were censored at the time of endpoint or loss of follow-up.

### Statistical analysis

The distributions of quantitative variables were assessed for normality. Continuous variables were expressed as the mean ± standard deviation (SD) (normally distributed variables) or median and interquartile range (IQR) (non-normally distributed variables). For continuous data, one-way analysis of variance was used if the variable was normally distributed; if not, Kruskal–Wallis tests were performed. Categorical data were expressed as frequencies and percentages (%) and compared by Pearson chi-squared tests or Fisher’s exact test, as appropriate. The probabilities of cumulative renal survival curves were generated by the Kaplan–Meier method, and the differences between curves were analyzed by a log-rank test. Univariate and multivariate Cox regression proportional hazards models were built to evaluate independent risk factors of renal progression. No violations of the Cox proportional hazards assumptions were detected. The results of Cox regression analyses were expressed as hazard ratios (HRs) with 95% confidence intervals (CIs). *P* values ≤ 0.05 were considered to indicate statistical significance with 95% CIs. Statistical Package for the Social Sciences (version 22.0, 2013, IBM SPSS Statistics for Windows, Armonk, NY: IBM Corp) and R software (version 3.5.1, 2013, R Foundation for Statistical Computing, Vienna, Austria. URL https://www.R-project.org/) were used for all statistical analyses.

## Results

The main characteristics of the included patients are shown in Table 1. A total of 330 cases were enrolled in this study, including 137 (41.5%) men and 193 (58.5%) women with a median age of 34.0 years and a median PLR of 120.4. Patients were followed for a median of 47.4 months.

**Table 1:** Clinical characteristics at the time of renal biopsy and outcomes of included patients categorized by the magnitude of PLR tertiles

Variables	All	PLR < 106	106 ≤ PLR ≤ 137	PLR > 137	*P*-value	Test statistic
*At the time of renal biopsy*						
Number of patients (*n*)	330	111	109	110		
Age (years, median [IQR])	34.0 [15]	36.0 [16]	34.0 [15]	33.0 [16]	0.598	1.030^a^
Male (*n*, %)	137(41.5)	49 (44.1)	44 (40.4)	44 (40.0)	0.787	0.479^b^
MAP (mmHg, mean (SD))	97.8 (13.7)	96.6 (14.1)	98.3 (13.1)	98.5 (13.9)	0.542	0.613^c^
WBC (× 10^9^/L, median [IQR])	6.79 [2.39]	7.06 [2.57]	6.79 [2.28]	6.34 [2.51]	0.163	3.624^a^
Lymphocytes (× 10^9^/L, median [IQR])	1.84 [0.75]	2.27 [0.70]	1.88 [0.55]	1.54 [0.51]	< 0.001	89.433^a^
Platelets (× 10^9^/L, median [IQR])	222.0 [83.5]	187.0 [77.0]	225.0 [73.0]	252.0 [77.2]	< 0.001	60.560^a^
Hemoglobin (g/L, mean (SD))	130.9 (20.2)	132.2 (18.2)	131.7 (21.7)	128.8 (20.5)	0.392	0.940^c^
Serum albumin (g/l, mean (SD))	39.3 (5.7)	39.8 (4.5)	39.3 (5.7)	38.9 (6.7)	0.440	0.869^c^
Scr (μmol/L, median [IQR])	83.5 [42.0]	83.0 [41.0]	86.0 [42.5]	82.0 [47.3]	0.799	0.449^a^
UA (μmol/L, mean (SD))	356.2 (99.2)	359.2 (90.4)	352.0 (94.6)	357.3 (112.2)	0.858	0.172^c^
eGFR (ml/min/1.73 m^2^, median [IQR])	89.5 [47.5]	92.6 [45.2]	83.7 [45.7]	88.9 [55.6]	0.778	0.503^a^
Proteinuria (g/24 h, median [IQR])	0.78 [1.34]	0.96 [1.00]	1.03 [1.52]	0.63 [1.57]	0.209	3.135^a^
*Follow-up and treatment*						
Follow-up (months, median [IQR])	47.4 [24.0]	49.6 [23.2]	49.1 [25.6]	43.9 [20.3]	0.016	8.332^a^
Composite endpoints (*n*, %)	27 (8.2)	6 (5.4)	6 (5.5)	15 (13.6)	0.038	6.535^b^
ESRD (*n*, %)	10 (3.0)	2 (1.8)	0 (0.0)	8 (7.3)	0.003	9.663^b^
Use of RAAS blockade (*n*, %)	151 (45.8)	48 (43.2)	57 (52.3)	46 (41.8)	0.241	2.847^b^
Use of IS (*n*, %)	236 (71.5)	76 (68.5)	80 (73.4)	80 (72.7)	0.679	0.774^b^

*PLR* platelet-to-lymphocyte ratio, *IQR* interquartile range, *SD* standard deviation, *MAP* mean arterial pressure, *WBC* white blood cell co nt, *Scr* serum creatinine, *UA* uric acid, *eGFR* estimated glomerular filtration rate, *RAAS* rein-angiotensin-aldosterone-system, *IS* immune suppression. Composite endpoint was eGFR decrease by 50% or ESRD. ESRD was defined as eGFR < 15 mL/min/1.73 m^2^, initiation of dialysis or renal transplantation

^a^Test statistic value for Kruskal–Wallis test (for non-normally distributed continuous data)

^b^Chi-square value for standard chi-squared test or Fisher’s exact test (for categorical data)

^c^*F*-ratio value for one-way ANOVA (normally distributed continuous data)

Patients were categorized by the magnitude of PLR tertiles into three groups: group 1 with PLR < 106 (*n* = 111), group 2 with 106 ≤ PLR ≤ 137 (*n* = 109) while group 3 with PLR > 137 (*n* = 110) (Table 1). There were no significant differences among the three groups in demographic characteristics, MAP, proteinuria, and renal function at baseline. And No significant differences were found among the three groups in the use of rein-angiotensin-aldosterone-system (RAAS) blockade and immune suppression (IS) treatments. Patients with higher PLR value had higher platelet count and lower lymphocyte count. There were 2 (0.6%) patients with a low level of platelet (< 100 × 10^9^/L) and 30 (9.1%) patients with a high level of platelet (> 300 × 10^9^/L). At the end of follow-up, 27 patients (8.2%) had reached the composite endpoints, including 17 patients with eGFR decrease by 50% and 10 patients with ESRD.

**Fig. 2:**
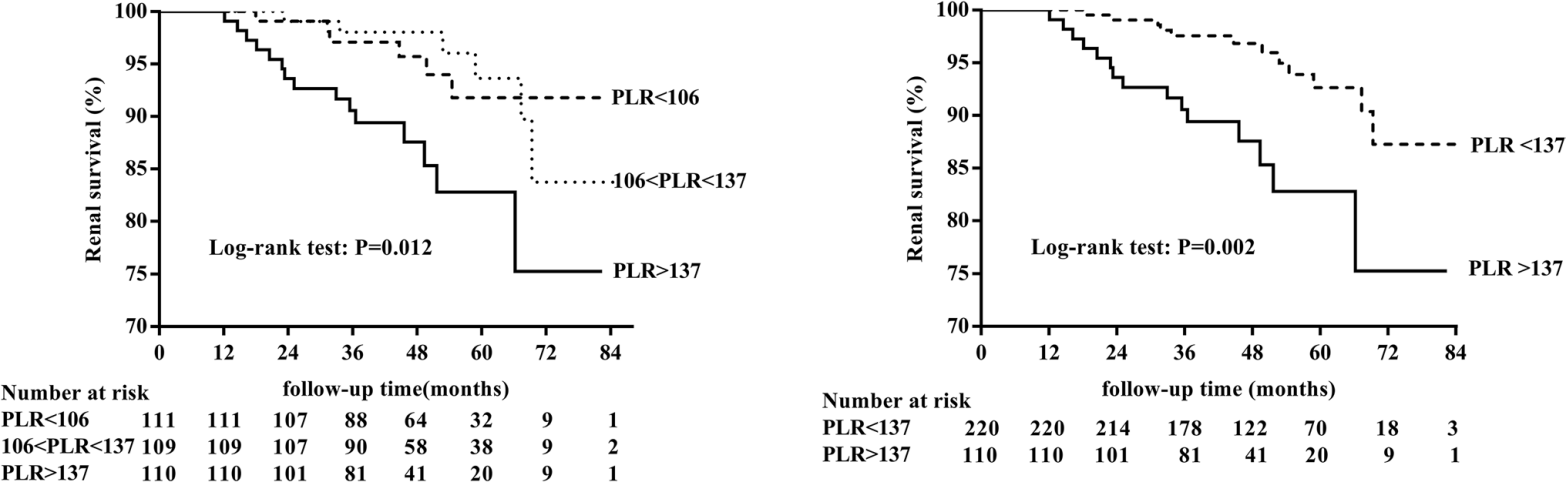
Renal survival curves for the patients with IgAN according to the platelet-to-lymphocyte ratio

The Kaplan–Meier curve showed the association of PLR with poor renal outcomes among IgAN patients (Fig. 2). As no significant difference in the Kaplan–Meier curve was seen between the first and the second tertile of the PLR group, we put the two groups together as PLR ≤ 137 group. Univariate analysis by Cox regression revealed that hemoglobin, serum albumin, Scr, UA, eGFR, and proteinuria at the time of renal biopsy and use of IS treatments were factors significantly associated with renal survival, which was defined by a status free of composite endpoints (Table 2). Besides, the lymphocyte count and platelet count were not associated with poor renal outcomes. When compared with group PLR ≤ 137, PLR > 137 was associated with a higher risk (HR 3.10; 95% CI 1.44–6.64; *P* = 0.004) of experiencing the poor renal outcomes. Moreover, after adjusted for age, sex, hemoglobin, serum albumin, Scr, UA, eGFR, proteinuria, and use of IS treatments, PLR > 137 (HR 2.79; 95% CI 1.08–7.26; *P* = 0.035) was still associated with poor renal outcomes among IgAN patients compared with PLR ≤ 137 (Table 3).

**Table 2:** Univariate Cox regression analyses of renal survival in patients with IgAN

Risk factor	Univariate		
	HR	95% CI	*P*-value
Age, years	1.00	0.96–1.04	0.962
Male	1.41	0.66–3.00	0.376
MAP, mmHg	1.02	0.99–1.05	0.128
WBC, × 10^9^/L	0.98	0.83–1.16	0.806
Lymphocytes, × 10^9^/L	0.57	0.28–1.19	0.134
Platelets, × 10^9^/L	1.00	0.99–1.01	0.471
PLR > 137	3.10	1.44–6.64	**0.004**
Hemoglobin, g/L	0.97	0.95–0.99	**0.001**
Serum albumin, g/l	0.92	0.88–0.97	**0.001**
Scr, μmol/L	1.04	1.03–1.05	** < 0.001**
UA, μmol/L	1.01	1.00–1.01	** < 0.001**
eGFR, ml/min/1.73 m^2^	0.92	0.90–0.95	** < 0.001**
Proteinuria, g/24 h	1.38	1.16–1.63	** < 0.001**
Use of RAAS blockade	0.59	0.27–1.30	0.190
Use of IS	0.23	0.11–0.51	** < 0.001**

*IgAN* Immunoglobulin A nephropathy, *HRs* hazard ratios, *CIs* confidence intervals, *PLR* platelet-to-lymphocyte ratio, *MAP* mean arterial pressure, *WBC* white blood cell count, *Scr* serum creatinine, *UA* uric acid, *eGFR* estimated glomerular filtration rate, *RAAS* rein-angiotensin-aldosterone-system, *IS* immune suppression

**Table 3:** Multivariate Cox regression analyses of renal survival in patients with IgAN

Risk factor	Multivariate		
	HR	95% CI	*P*-value
Age, years	0.93	0.88–0.99	**0.019**
Male	0.67	0.13–1.01	0.637
PLR > 137	2.79	1.08–7.26	**0.035**
Hemoglobin, g/L	0.98	0.96–1.01	0.199
Serum albumin, g/l	0.97	0.89–1.07	0.582
Scr, μmol/L	1.00	0.95–1.05	0.962
UA, μmol/L	1.00	1.00–1.01	0.925
eGFR, ml/min/1.73 m^2^	0.94	0.87–1.02	0.131
Proteinuria, g/24 h	1.20	0.86–1.68	0.284
Use of IS	0.17	0.06–0.52	**0.002**

*IgAN* Immunoglobulin A nephropathy, *HRs* hazard ratios, *CIs* confidence intervals, *PLR* platelet-to-lymphocyte ratio*, Scr* serum creatinine, *UA* uric acid, *eGFR* estimated glomerular filtration rate, *IS* immune suppression

**Fig. 3:**
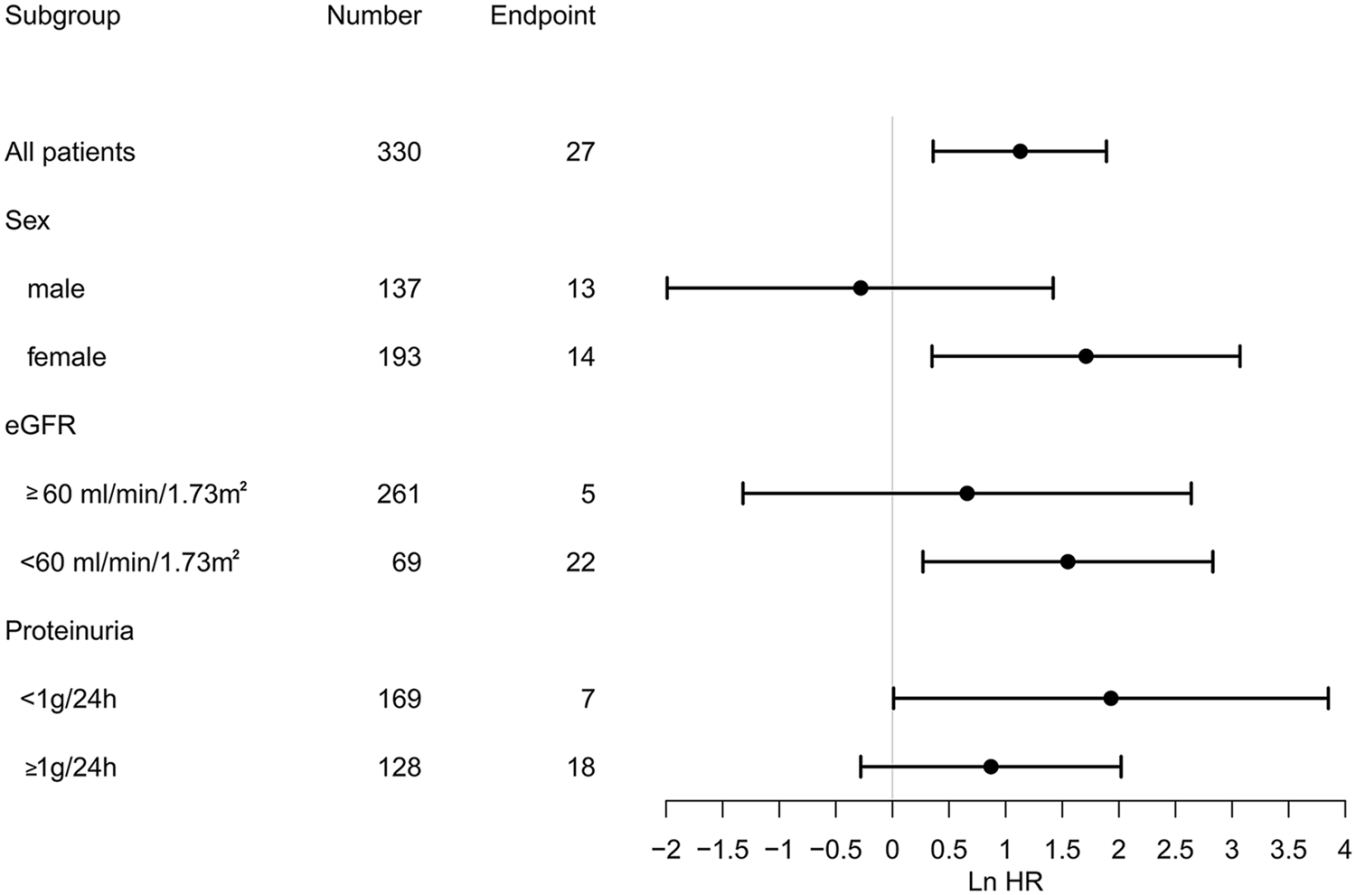
Subgroup analyses about the association of platelet-to-lymphocyte ratio and renal survival according to sex, baseline eGFR, and baseline proteinuria for the patients with IgAN

To be more precise, we also analyzed the renal survival of patients according to the sex, baseline eGFR, and proteinuria categories. As shown in Fig. 3, among female patients, PLR > 137 was associated with poor renal outcomes compared with PLR ≤ 137 after adjusted for age, hemoglobin, serum albumin, Scr, UA, eGFR, proteinuria, and use of IS treatments. We also found that patients with baseline eGFR lower than 60 mL/min/1.73 m^2^ had a significantly better renal survival in patients with PLR ≤ 137 than PLR > 137. While no significant difference was observed between PLR ≤ 137 and PLR > 137 groups in male patients or patients whose baseline eGFR higher than 60 mL/min/1.73 m^2^, baseline proteinuria higher than 1 g/24 h or lower than 1 g/24 h.

## Discussion

Although the platelet count and lymphocyte count are routinely evaluated under simple laboratory conditions among the patients with IgAN, the clinical significance of PLR in the IgAN process remains unclear. Our study evaluated the relationship between PLR and the renal regression of IgAN and demonstrated that PLR > 137 were independent prognostic factors for the long-term renal survival of patients with IgAN.

The advantage of the PLR is that it reflects the condition of patients in both inflammation and thrombosis pathways. It may be more valuable than either platelet or lymphocyte count alone. In our study, the mean value and corresponding 95% reference interval for the PLR in IgAN patients was 125.8 (63.3–227.9), which was close to the PLR value in the general population reported in a population-based prospective cohort study: 120 (61–239) [23]. Most studies showed that elevated PLR value played a predictive role in several diseases [12, 24–27]. Our study found the same outcomes in IgAN. As we all know, platelets and lymphocytes are derived from the same hematopoietic stem cells and the PLR should be kept constant for homeostasis [28, 29]. Higher PLR condition means relatively high platelets and low lymphocytes. Higher platelet count may reflect increased thrombocyte activation, which contributes to increased inflammation and thrombocytosis, and thereby result in adverse renal outcomes [30, 31]. Lymphocytopenia, which reveals depression of innate cellular immunity, may be induced by the systemic inflammatory response, and be responsible for an inadequate immunologic reaction and a weakened defense [32, 33]. Considering platelets and lymphocytes together, an elevated PLR might predict the bad body condition and weakening inflammatory response in those IgAN patients with poorer renal outcomes.

After Stratified analysis, we also found that the prognostic value of PLR remained the same for female patients or patients with eGFR less than 60 mL/min/1.73 m^2^. As we all know, females tend to mount stronger inflammatory responses than males [34–38], so the inflammation indicator, PLR, was more sensitive in female patients with IgAN. And for those IgAN patients with eGFR less than 60 mL/min/1.73 m^2^, they may have increased serum levels of inflammatory mediators and were more susceptible to inflammation-related vascular dysfunction and cardiovascular risk compared with healthy controls [39, 40]. Turkmen et al. [24] carried out a cross-sectional study involving 62 ESRD patients prompted that a simple calculation of PLR can predict inflammation in ESRD patients. These may suggest that those IgAN patients with worse renal function have been in a long-term inflammatory state, so the inflammation factors occupy a larger proportion in the progression to renal failure.

Besides the PLR, hemoglobin, serum albumin, Scr, UA, eGFR, proteinuria, and use of IS treatments were also factors significantly associated with renal survival in the present study. Several clinical indicators such as renal function and proteinuria at biopsy are demonstrated to be associated with the renal outcome of IgAN [7–9, 41, 42]. Our study also found that the use of IS therapy was associated with better renal outcomes among IgAN patients. IS therapy was a common treatment for immune-mediated kidney disease, however, the role of the use of IS therapy in IgAN was still controversial [43–47]. Larger prospective studies with more patients and longer follow-up time were needed to assess the effect of IS therapy in IgAN.

This study had several limitations. The potential limitation of the present study was that it was a retrospective, single-center study with a relatively small sample size of patients and a low number of endpoints reached. Thus, larger prospective studies, multi-site studies with patients of various ethnicities are needed to confirm these preliminary results. Another limitation was that we measured the blood pressure with two different devices with two different measurement errors. Due to the nature of the retrospective cohort study, we are not able to remeasure the blood pressure and recollect information which might result in recall or misclassification bias. Also, besides PLR, there were some other markers of inflammation, such as CRP, serum iron, ferritin [48], which should be tested and taken into consideration in our future study.

In conclusion, we demonstrated that PLR > 137 was independent prognostic factors for the long-term renal survival of IgAN. Clinicians can pay more attention to the PLR value in the management of IgAN patients, especially for those female patients or whose eGFR lower than 60 mL/min/1.73 m^2^.

## Abbreviations

PLR: Platelet-to-lymphocyte ratio
IgAN: Immunoglobulin A nephropathy
ESRD: End-stage renal disease
SBP: Systolic blood pressure
DBP: Diastolic blood pressure
MAP: Mean arterial pressure
WBC: White blood cell count
Scr: Serum creatinine
UA: Uric acid
eGFR: Estimated glomerular filtration rate
MDRD: Modification of Diet in Renal Disease Study
SD: Standard deviation
IQR: Interquartile range
HRs: Hazard ratios
CIs: Confidence intervals
RAAS: Rein-angiotensin-aldosterone-system
IS: Immune suppression
